# Effect of Different Pre-Growth Temperatures on the Survival Kinetics of *Salmonella enterica* and *Listeria monocytogenes* in Fresh-Cut Salad during Refrigerated Storage

**DOI:** 10.3390/foods12234287

**Published:** 2023-11-28

**Authors:** Avninder Kaur, Veerachandra Yemmireddy

**Affiliations:** 1 School of Integrative Biological and Chemical Sciences, The University of Texas Rio Grande Valley, 1201 W University Dr, Edinburg, TX 78539, USA; avninder.kaur01@utrgv.edu; 2 School of Earth, Environmental and Marine Sciences, The University of Texas Rio Grande Valley, 1201 W University Dr, Edinburg, TX 78539, USA

**Keywords:** fresh-cut salad, temperature stress, foodborne pathogens, storage, survival kinetics

## Abstract

The effect of the pre-growth temperature of bacterial cultures on their subsequent survival kinetics in fresh-cut produce during refrigerated storage was investigated in this study. Three-strain cocktails of *Listeria monocytogenes* and *Salmonella enterica*, cultured at different growth temperatures (4, 21, and 37 °C) were inoculated on fresh-cut mixed salad and on individual produce in the mixed salad. The inoculated samples were stored at 4 °C and 80 ± 2% relative humidity (RH) for up to 72 h and the growth, survival, or death kinetics were determined at regular intervals. The results indicate that depending upon the type of pathogen tested, the pre-growth temperature(s) and the type of produce showed a significant (*p* ≤ 0.05) effect on the survival kinetics. Among the tested produce, mixed salad showed the highest reduction in *L. monocytogenes* pre-grown at 37 °C (1.33 log CFU/g) followed by red cabbage (0.56 log CFU/g), iceberg lettuce (0.52 log CFU/g), and carrot (−0.62 log CFU/g), after 72 h, respectively. In the case of *Salmonella*, carrot showed the highest reduction (1.07 log CFU/g for 37 °C pre-grown culture) followed by mixed salad (0.78 log CFU/g for 37 °C pre-grown culture), cabbage (0.76 log CFU/g for 21 °C pre-grown culture), and lettuce (0.65 log CFU/g for 4 °C pre-grown culture), respectively. Among the tested ComBase predictive models, the Baranyi–Roberts model better fitted the experimental data. These findings indicate that the appropriate selection of pre-growth environmental conditions is critical to better understand the kinetics of foodborne pathogens.

## 1. Introduction

Leafy greens are a vital source of vitamins, minerals, dietary fiber, and phytonutrients and are attributed to lowering the risk of several chronic diseases such as diabetes, obesity, cardiovascular disease, hypertension, and cancer [1,2,3]. In the recent past, the demand for fresh, convenient, preservative-free, minimally processed, and health-promoting foods such as fresh-cut fruits and ready-to-eat (RTE) mixed salads [4,5,6] has increased. However, fresh-cut produce is prone to rapid deterioration due to moisture loss, accelerated enzymatic activity, and microbial proliferation [7,8,9]. Moreover, leafy greens have been implicated in several foodborne illness outbreaks [10,11,12]. Foodborne bacterial pathogens such as *Listeria monocytogenes* [13,14,15] and *Salmonella* [11,16,17] are associated with multiple illness outbreaks in fresh and fresh-cut produce. 

*Listeria monocytogenes* is of particular concern in RTE salad mix due to its ability to grow in aerobic or anaerobic environments as well as at refrigeration temperatures [18,19,20]. In 2021, a multi-state outbreak of packaged salads was attributed to a contamination of *L. monocytogenes* [21]. Many studies have determined the survival kinetics of foodborne pathogens in fresh produce and fresh-cut salad during storage under different conditions, and found that depending upon storage conditions, few commodities supported the growth and proliferation of pathogens, while others were not very supportive. Studies have revealed that nutrients released from the exposed cytoplasm of cut fresh produce enable the proliferation and growth of foodborne pathogens [22,23]. A study by Waitt et al. [24] reported that a storage temperature of 12 °C increased *Salmonella* levels on lettuce roots and leaves, while 4 °C did not support the growth. Similarly, Lee et al. [25] reported that populations of *E. coli* O157:H7, *S. enterica*, and *L. monocytogenes* increased rapidly in red cabbage juice when stored at 10 °C for 72 h. A few studies have reported an increase in levels of *L. monocytogenes* by 1.5 (at 8 °C) and 2.3 (at 12 °C) log CFU/g in iceberg lettuce [26]; 3.02 log CFU/g in salad mixes of lettuce, cucumber, tomato, carrot, and red cabbage [27]; 1.85 (arugula), 0.88 (spinach), 0.51 (green salad), 0.49 (escarole), 0.30 (collard green), and 0.21 (watercress) log CFU/g at 7 °C [28]; and 1.4 (broccoli) and 1.6 (cauliflower) log CFU/g at 4 °C [29]; while other studies have reported a reduction in *L. monocytogenes* by 3 (Arabic salad at 4 °C), 2.3 (tahini salad at 4 °C), 3.3 (basil at 21 °C), 2.4 (cilantro at 21 °C), 2.6 (dill at 21 °C), 3.2 (parsley at 21 °C), and 3.61 (carrots at 7 °C) log CFU/g during storage [28,30,31]. Huang et al. [32] reported a decrease in *S. enterica* levels on cantaloupe, watermelon, and radish when stored at 4 °C for 7 days, while *S. enterica* levels on pineapple did not change under the same conditions. López-Gálvez et al. [33] and Singh and Yemmireddy [34] reported the effect of storage relative humidity on the survival characteristics of S. *enterica* on lettuce and fresh-cut papaya, respectively. Studies have also reported that the presence of antilisterial phytoalexins in carrots inhibited the growth of *L. monocytogenes* [14,35,36]. Huang et al. (2019) [32] concluded that cut radish hindered bacterial growth due to its antibacterial properties, whereas its juice supported the growth of *Salmonella* and *L. monocytogenes*. This shows that factors such as the type of pathogen, type of fresh produce, storage time, temperature, and relative humidity have a profound effect on the growth, survival, and death kinetics of foodborne bacterial pathogens [37,38,39].

A careful analysis of the past research work on fresh-cut produce reveals two important aspects: (i) the evaluation of microbial growth, survival, and death kinetics using cells that were grown or cultured under ideal laboratory conditions; (ii) the development, validation, and verification of interventions based on the perceived kinetics data from an earlier point. These scenarios are far from real-life conditions where microbes are constantly exposed to various environmental stresses such as stable and dynamic temperatures, relative humidity, pH, desiccation, salt stress, etc. These stressors either increase or decrease the susceptibility of respective organisms. Often the importance of key methodological aspects while designing post-harvest storage studies such as bacterial-strain characteristics, inoculum preparation, recovery, and enumeration or detection procedures is overlooked and lacks uniformity. Bolten et al. [40] emphasized these aspects in their scoping review on the population dynamics of *Listeria* spp., *Salmonella* spp., and *E. coli* on fresh produce in pre-harvest and post-harvest environments. Limited information exists on the effect of pre-growth environmental stresses on the survival kinetics of foodborne pathogens on food matrices. Harrand et al. [41] observed the effect of pre-growth conditions on the growth of *Listeria and E. coli* on select fresh fruits and vegetables. Similarly, *L. monocytogenes* when subjected to pre-growth stresses showed less reductions upon exposure to different chemical treatments than *S. enterica* and *E. coli* O157:H7 [34]. However, the effect of different pre-growth conditions on the fate of *L. monocytogenes* and *Salmonella enterica* in fresh-cut salad in a simulated real-world storage scenario is not well understood. Particularly, in pre-harvest, post-harvest, and distribution stages, fresh produce and associated microflora may be subject to varying temperature stresses. Thus, the main objective of this study was to determine the effect of pre-growth temperature on the growth, survival, and/or death kinetics of *L. monocytogenes* and *Salmonella enterica* in fresh-cut salad vegetables during simulated retail refrigerated storage. This information would help to better understand the population dynamics of foodborne bacterial pathogens and develop more robust food safety intervention strategies. 

## 2. Methodology 

### 2.1. Selection of Bacterial Strains

A three-strain cocktail of *L. monocytogenes* (i. F8027, serotype 4b, celery isolate; ii. 101 M, serotype 4b, beef-associated outbreak isolate; and iii. F8385, serotype 1/2b, carrot isolate) and a three-strain cocktail of nalidixic-acid-adopted *Salmonella enterica* (i. Newport 11,590 K, beef isolate; ii. Poona A3279, cantaloupe-associated outbreak; and iii. St. Paul E20081236, jalapeno outbreak) were used as test pathogens. The cocktail inoculums of individual bacterial types were chosen over individual strains to identify population dynamics in worst-case scenarios. All the *Salmonella* strains were modified for antibiotic resistance with 0.05 g L^−1^ nalidixic acid (NA; Fisher Scientific, Fair Lawn, NJ, USA) using a continuous stepwise exposure [42]. The stocks of all the strains containing tryptic soy broth (TSB, Hardy diagnostics, Santa Maria, CA, USA) and 25% glycerol (*wt*/*wt*) were stored at −80 °C. The frozen stocks of selected strains were thawed at room temperature in a biosafety cabinet and activated by transferring a loopful of inoculum into 10 mL tryptic soy broth with 0.1% yeast extract (TSBY) for *L. monocytogenes* and 10 mL of tryptic soy broth with nalidixic acid (TSBN). These cultures were then grown under different temperature conditions as discussed below. 

### 2.2. Growing Bacterial Cultures and Inoculum Preparation

Individual strains of selected bacteria were grown under three different temperatures, 37, 21, and 4 °C, to represent different real-world scenarios. Briefly, (i) strains of *S. enterica* and *L. monocytogenes* were grown in TSBN and TSBY, respectively, at 37 °C and 200 rpm in a shaker incubator for up to 18 h. This condition is referred to as the common laboratory growth conditions followed in many studies. (ii) Cells were grown at 21 °C for up to 36 ± 2 h reflecting growth at room temperature, and (iii) cells were grown at 37 °C for 8 h to increase the cell numbers initially and then adopted at 4 °C to reflect refrigerated storage for up to 40 h to achieve the desired working concentration of 10^7−8^ CFU/mL bacterial cells. Afterward, an equal volume (5 mL) of each individual strain in each category was mixed to prepare the three-strain cocktails, which were harvested by centrifuging at 4000× *g* for 10 min (5920R, Eppendorf^TM^, Hamburg, Germany). The resultant pellet(s) was/were resuspended in 10 mL of 0.1% buffered peptone water (BPW, Hardy diagnostics, Santa Maria, CA, USA), and serial dilutions were performed in BPW to achieve a working concentration of 10^6^ CFU/mL. Cell concentrations were confirmed by plating 100 µL portions of the appropriate serial dilutions on selective media Oxford agar (*Listeria*) and xylose lysine deoxycholate supplemented with 0.05 g L^−1^ nalidixic acid agar (XLDN; *Salmonella*) plates and incubation at 37 °C for 24 h.

### 2.3. Sample Preparation, Inoculation, and Storage 

**Figure 1:**
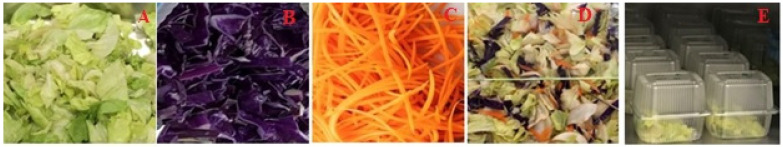
Samples of fresh-cut produce: (**A**) iceberg lettuce, (**B**) red cabbage, (**C**) grated carrot, and (**D**) mixed salad. (**E**) Clam-shell containers stored in an environmental chamber at 4 °C and 80 ± 2% RH.

Fresh iceberg lettuce (*Lactuca sativa* cv capitata), red cabbage (*Brassica oleracea* cv capitata f. rubra), and carrot (*Daucus carota*) were purchased from the local retail store (McAllen, TX, USA) and stored at 4 °C for use in experiments within 24 h. After removing the outer 2–3 layers of lettuce and cabbage, all the vegetables were thoroughly rinsed with deionized water and air-dried in a biosafety cabinet at room temperature. The dried lettuce and cabbage were aseptically chopped to a desirable size (5 cm length × 2 cm width), and the carrots were grated as shown in Figure 1. A 50 g sample(s) weighed in stomacher bags (Whirl-Pak™, Pleasant Prairie, WI, USA) and an aliquot of 5 mL of inoculum was/were added to each individual bag. The bags were thoroughly mixed by shaking and gentle massaging for about 2 min to achieve a uniform distribution of the inoculum of each sample. The individual batches of inoculated samples were pooled together in a sterile tray (250 g total sample size for each produce item) and mixed again using a sterile spatula. For the mixed-salad samples, 70:20:10 (wt %) of iceberg lettuce, red cabbage, and carrot were mixed and then followed the inoculation procedure as described above. All the samples were air-dried in a biosafety cabinet for 1 h, and portions of 25 g were weighed in 20 Oz clam-shell boxes (Dart, Troy, MI, USA) and then stored in an environmental chamber (Forma 3900 Series, Thermo Scientific™, Waltham, MA, USA) at 4 °C and 80 ± 2% RH (Figure 1) to simulate retail storage conditions. Appropriate control samples inoculated with just sterile deionized water and subjected to the same treatment conditions were also included. Samples were collected at regular intervals (0, 12, 24, 48, and 72 h), and the viability of test pathogens (both on the treatment and control samples) and spoilage organisms (only on control samples) were enumerated. 

### 2.4. Enumeration

For each sampling time, an aliquot of samples (10 g) was collected from the clam-shell containers and transferred to Whirl-pak™ bags containing 90 mL (1:9 *w*/*v*) of 0.1% BPW. These samples were homogenized using a stomacher (Model 400 Seward™, Seward, NE, USA) for 2 min at 200 rpm. Appropriate serial dilutions of the samples were prepared in BPW and plated on Oxford agar (for *L. monocytogenes*) and XLDN (for *Salmonella*) in duplicates. The plates were incubated at 37 °C for 24 ± 2 h, and the surviving cells were reported as log CFU/g. The rate of spoilage during storage caused by yeast and mold and the aerobic plate count (APC), were determined using 3M Petrifilms (3M™, St. Paul, MN, USA) following the manufacturer’s instructions. 

### 2.5. Modeling Survival Kinetics 

ComBase predictive models were assessed to determine the models that best fit the experimental data using the Online DMFit tool. The data were analyzed using Baranyi and Roberts [43] and linear models to determine the predictive power of these models. 

### 2.6. Statistical Analysis

All the experiments were conducted in triplicate, and duplicate samples were included in each replicated experiment. The CFU data were transformed into log CFU/g and arranged to conduct statistical analysis by comparing the results within the same commodity and across different commodities during storage for different pre-growth temperature conditions. Data were analyzed by the analysis of variance (ANOVA) procedure using SPSS™ (Version 28, IBM^®^, Armonk, NY, USA). A Tukey’s multiple comparison test was performed to determine the mean differences. All the tests were performed with a 0.05 level of significance.

## 3. Results and Discussion

### 3.1. Effect of Pre-Growth Temperature on the Survival Kinetics of L. monocytogenes 

**Figure 2:**
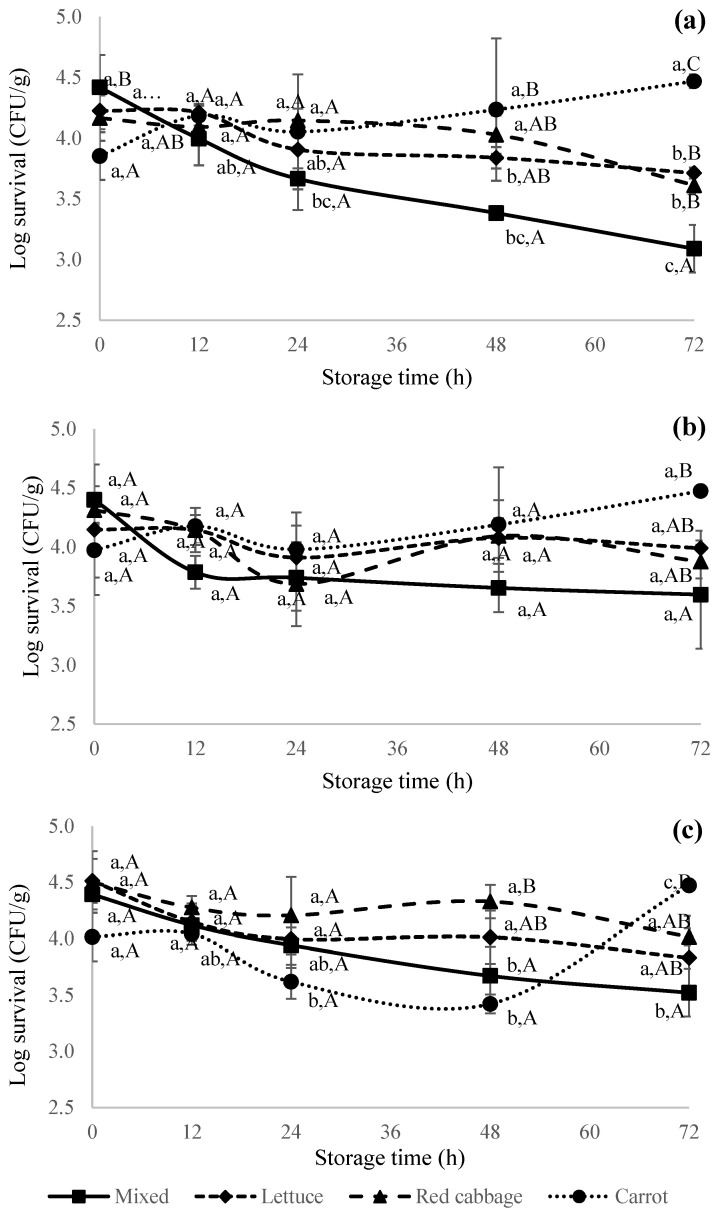
Survival kinetics of *Listeria monocytogenes* on iceberg lettuce (♦), red cabbage (▲), shredded carrot (•), and mixed salad (■) when pre-cultured at (**a**) 37 °C, (**b**) 21 °C, and (**c**) 4 °C. Lower-case letters indicate a significant difference within the same produce during the storage period, whereas upper-case letters indicate a significant difference across different produce at a single time point. *L. monocytogenes* was not detected on control samples; hence, they are not shown in the figure above.

Figure 2 shows the survival kinetics of *L. monocytogenes* that were pre-cultured at 37 (Figure 2a), 21 (Figure 2b), and 4 (Figure 2c) °C, respectively, and subsequently inoculated on different types of fresh-cut produce (i.e., iceberg lettuce, red cabbage, and grated carrot) and mixed salad during simulated retail storage for 72 h at 4 °C and 80 ± 2% RH. The results indicate that both factors, the culture pre-growth temperature and the type of produce, have a significant (*p* ≤ 0.05) effect on the survival kinetics of *L. monocytogenes*. When *L. monocytogenes* was pre-cultured in nutrient-rich media under normal laboratory growth conditions at 37 °C and subsequently inoculated on carrot, it showed a slight increase in its levels (*p* > 0.05), while the other produce items, lettuce, red cabbage, and mixed salad, showed a decrease in its levels (*p* ≤ 0.05) at the end of 72 h storage (Figure 2a). At the end of refrigerated storage, the levels of *L. monocytogenes* on the mixed salad were significantly (*p* ≤ 0.05) lower when compared to red cabbage or iceberg lettuce and grated carrot alone. A decrease of 1.33 log CFU/g was observed in the case of mixed salad followed by 0.56 log CFU/g (red cabbage) and 0.52 log CFU/g (iceberg lettuce), respectively. However, no significant (*p* > 0.05) change in the levels of *L. monocytogenes* was observed in the case of grated carrot. These findings can be attributed to two main factors: (i) the ability of *L. monocytogenes* to survive at refrigerated temperatures and (ii) the physical condition and/or chemical properties of the tested produce and its interaction with *L. monocytogenes*. It is understandable that *L. monocytogenes* is able to survive at low storage temperatures due to its psychotropic properties. However, the type of produce and storage time were found to be rate limiting factors. 

Most previous studies have used bacteria that were cultured at ideal laboratory growth conditions of 37 °C in nutrient-rich media. However, increasing evidence shows that pre-growth environmental conditions affect the tolerance, growth, and survival kinetics of pathogens [34,41]. Thus, we investigated the survival kinetics of *L. monocytogenes* when subjected to different (21 and 4 °C) pre-growth temperatures than the earlier-mentioned 37 °C. In our study, relative humidity was also included as an additional factor to best simulate the survival kinetics of *L. monocytogenes* in tested produce during short-term retail or consumer storage. When using the *L. monocytogenes* cells that were pre-cultured at 21 °C, irrespective of the type of produce, no significant (*p* > 0.05) change in their levels was observed during storage (Figure 2b). Non-significant fluctuations across all the produce types were noticed for the first 24 h (Figure 2a,b). This can be attributed to the adjustment of cells to new environmental conditions during subsequent storage at the refrigerated temperature (4 °C) compared to their prior culturing and growth temperatures of 37 and 21 °C, respectively. Further decreasing the culture growth temperature to 4 °C and the subsequent inoculation on different produce showed almost a similar trend as cells that were pre-cultured at 21 °C (Figure 2c). Thus, *L. monocytogenes* which was pre-cultured at 37, 21, and 4 °C showed an overall reduction of 1.33, 0.8, and 0.87 log CFU/cm^2^, respectively, on mixed salads, while on grated carrot, it showed a growth of 0.62, 050, and 0.45 log CFU/cm^2^, respectively. These respective reductions on mixed salad and/or growth on carrot significantly differ from other tested produce (i.e., lettuce and red cabbage) under the same test conditions. However, the changes were not significantly different within the same produce (mixed salad/carrot) across different pre-growth conditions. One possible reason for the growth on carrots could be due to the oxidation of phenolic compounds in shredded carrots which reduced its potential antimicrobial activity. The antioxidant activity of fresh produce can also be reduced by its direct oxidation by polyphenol oxidase and peroxidase enzymes [44].

Studies have reported that minimally processed fresh-cut or grated vegetables facilitate the attachment and further survival of pathogens due to the release of nutrients to nourish the attached bacteria, which aids their proliferation [19,23,45] or death due to the release of antimicrobial compounds such as phytochemicals and the presence of natural microbiota [46,47,48]. Carrasco et al. [49] reported a 3 log CFU/g increase in an *L. monocytogenes* population in RTE lettuce after storage at 13 °C for 7 days. On the other hand, studies have reported that cut red cabbage at low temperatures did not support the growth of *L. monocytogenes* [50] and *Salmonella* [14], respectively. Red cabbage is a rich source of antioxidants such as anthocyanins, glucosinolates, and sulfur compounds such as methyl methanethiosulfinate, known to have inhibitory effects on foodborne pathogens [51,52,53,54]. Similarly, the inhibitory effect of grated or cut carrots on *L. monocytogenes* [25,36] and *S. typhimurium* [55] was reported. Additionally, phenolic phytoalexins such as 6-methoxymellein present in carrot [55] and red cabbage have the ability to change the permeability of microbial cell membranes, which can result in the leakage of cellular content, the denaturation or inhibition of crucial metabolic enzymes, and ultimately death [53,56]. In fresh carrot juice, carotol (20.20%) and sabinene (12.80%) were found to be major constituents exhibiting inhibitory effects on Gram-positive and Gram-negative bacteria [35]. A study by Demirdoven et al. [51] reported the extracted total anthocyanin value as 593 mg/L and total phenolic acid content as 16,320 mg/L in red cabbage samples. A similar metabolic-profiling study reported a total phenolic content (majorly composed of rutin, quercetin, and catechin) of 1114.60 μg/g and sixteen anthocyanins in red Chinese cabbage showing a positive correlation to pathogenic growth inhibition [57]. In the current study, no significant difference in the levels of *L. monocytogenes* was observed between iceberg lettuce and red cabbage (Figure 2). This can be potentially attributed to limited or no exposure to the available phytochemicals in red cabbage and grated carrot within the tested short-term retail storage period to potentially exert a significant reduction as noticed in previous studies. As per our observation, inoculated fresh-cut red cabbage (Figure 1B) looked dry in appearance after air-drying and during refrigerated storage with no signs of damage and exposure to intracellular components. The level of reductions that were observed in mixed salad when cells were pre-grown at 37 °C was not maintained when cells were pre-grown at temperatures of 21 and 4 °C. This shows that pre-growth environmental conditions in addition to time and temperature are critical factors that affect the growth, survival, and/or death kinetics of *L. monocytogenes* on fresh-cut salad during refrigerated storage. Depending upon the type of produce, *L. monocytogenes* was found to better survive when pre-growth culture temperatures were 4 or 21 °C compared to the commonly used culture growth temperature of 37 °C. To further understand the effect of the type of pathogen and its dependence on the culture growth temperature, follow-up studies were conducted on *S. enterica*. 

### 3.2. Effect of Pre-Growth Temperature on the Survival Kinetics of S. enterica

**Figure 3:**
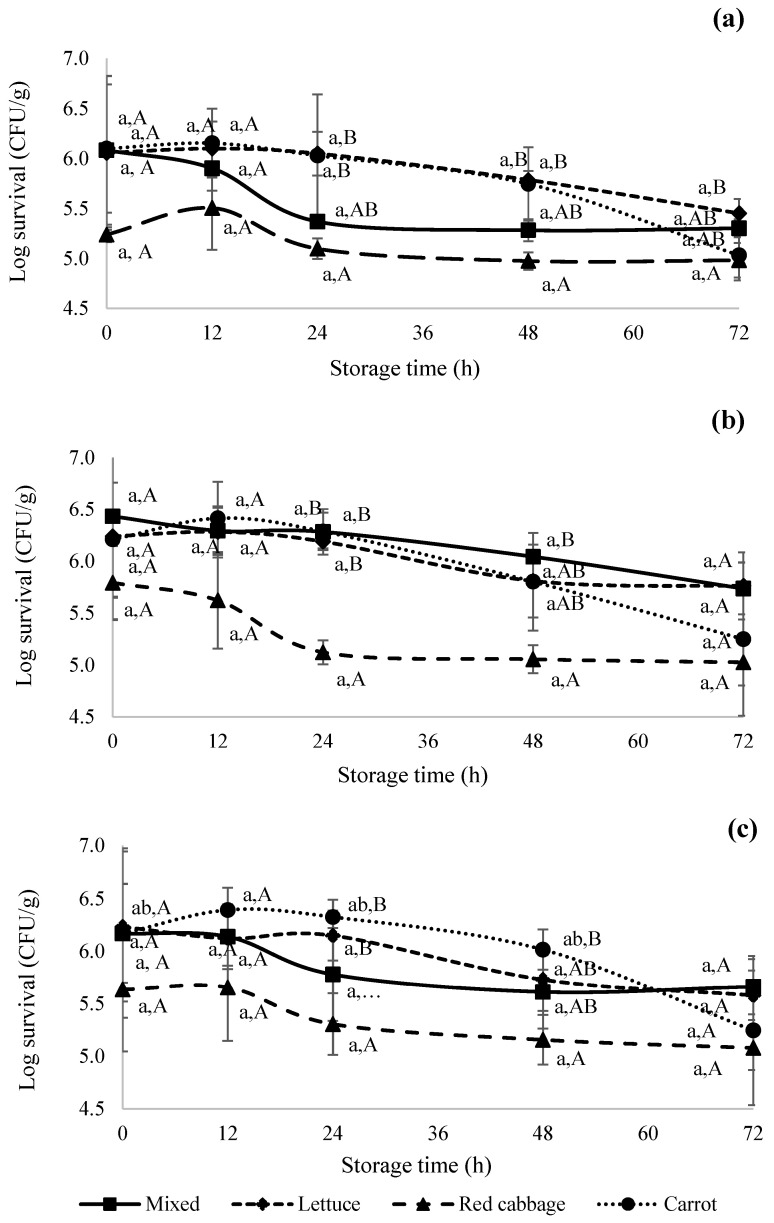
Survival kinetics of *Salmonella enterica* on iceberg lettuce (♦), red cabbage (▲), shredded carrot (•), and mixed salad (■) when pre-cultured at (**a**) 37 °C, (**b**) 21 °C, and (**c**) 4 °C. Lower-case letters indicate a significant difference within the same produce during the storage period, whereas upper-case letters indicate a significant difference across different produce at a single time point. *S. enterica* was not detected on control samples; hence, they are not shown in the figure above.

Figure 3 shows the survival kinetics of *S. enterica* pre-cultured at 37 (Figure 3a), 21 (Figure 3b), and 4 (Figure 3c) °C, respectively, and subsequently inoculated on different types of produce during simulated retail storage at 4 °C and 80% RH for up to 72 h. Unlike *L. monocytogenes*, pre-growth temperature and the type of produce did not show a significant (*p* > 0.05) effect on the survival kinetics of *Salmonella*. Within the same produce type, *Salmonella* levels neither increased nor decreased during the storage time (Figure 3a–c). In general, red cabbage showed the lowest survival rate when compared to other tested produce. A study by Vandamm et al. [58] reported the −0.15 log CFU/g growth of *Salmonella* on cut celery stored in containers at 4 °C for 7 days. Harrand et al. [41,59] reported that pre-growth conditions such as 37 °C, 21 °C, low pH, high salt, and a minimal medium had varying effects on *S. enterica*, *E. coli,* and *Listeria* when tested on lettuce, cantaloupe rind, and tomatoes. As per their study, depending upon the pre-growth condition tested, *E. coli* showed an overall decline (<1 log) over a 7-day period, while *S. enterica* and *Listeria* showed regrowth after an initial reduction. Similarly, the findings of our study indicate that *S. enterica* and *L. monocytogenes* levels showed a slight decline initially for certain produce items and afterward either survived or declined (<1.5 log) during the short-term refrigerated storage (Figure 2 and Figure 3). This could potentially be attributed to a range of adaptive mechanisms of the bacteria to adjust to different environmental conditions. Thakur et al. [60] explained the possible mechanisms of adaptations by differential gene expression in pathogens under adverse environmental conditions. The presence of transcription factors, such as sigma factors, is responsible for the transcription of genes involved in the resistance to diverse stresses [61]. A study reported that *Salmonella* Enteritidis upregulates the genes involved in the resistance to heat (rpoH, uspB, and htrA), cold (cspA, cspC, and csdA), acid (SEN1564A and cfa), and salt (proP, proV, and osmW) [62]. However, further studies need to be conducted to understand the possibility of these mechanisms under the conditions tested in the current study. 

### 3.3. Determination of Aerobic Plate Count (APC), Yeast, and Mold during Storage 

Table 1 shows the APC and yeast and mold counts on the iceberg lettuce, red cabbage, grated carrot, and mixed-salad samples during refrigerated storage for 72 h. No significant (*p* > 0.05) change in the APC was observed during storage within and across the different produce tested. Red cabbage was comparatively found to have a lower APC, though it was not statistically different from the other produce at the end of 72 h. A study by Nagarajan et al. [63] reported low APC levels in red cabbage, and this was attributed to the activity of antimicrobial polyphenolic oxidases. Yeast counts were found to be higher than the APC. Similarly, a higher mold count was observed in shredded carrot (1.13 log CFU/g) and mixed salad (1.04 log CFU/g) by the end of the storage period. Similarly, an increase in yeast (4.1 log CFU/g) and mold (3.85–6.7 log CFU/g) count was reported on spinach leaves when stored at 5 °C [64]. As per our observation, the tested produce maintained its freshness during the storage period, and no visual deterioration and quality changes were observed. As such, the effect of naturally present microbiota on the survival kinetics of pathogens in the tested produce is beyond the scope of the current study; however, it is a key factor that should not be overlooked. Typically, fruits and vegetables carry naturally present microbiota such as *Pseudomonas* spp., *Erwinia herbicola, Flavobacterium*, *Xanthomonas*, and *Enterobacter,* as well as various yeasts and molds [22,65]. Lactic-acid bacteria such as *Lactobacillus* spp. and other natural microbiota can exhibit antagonistic properties against pathogens [66,67]. In our study, we tried to deduce the potential relationship between pathogenic (*L. monocytogenes*, *S. enterica*) and spoilage organisms (APC, Y&M), but the data were just not sufficient to provide any conclusive details.

### 3.4. Modeling the Survival Kinetics of L. monocytogenes and S. enterica 

Table 2 shows the parameter estimates of *L. monocytogenes* and *S*. *enterica* when pre-cultured at different temperatures and subsequently inoculated onto fresh-cut produce including mixed fresh-cut salad during refrigerated storage. The experimental data were tested to check the goodness of fit using ComBase predictive models and the DMFit online tool. Among the different models in the tool, both the Baranyi and Roberts and linear models fitted the data. However, the Baranyi and Roberts model (with no lag) in the majority of cases better fitted the data (R^2^ ranging from 0.054 to 0.967 for *L. monocytogenes* and 0.194 to 0.994 for *S. enterica*) with a lower standard error of fit when compared to linear models (Supplementary Materials). Under the tested conditions, the maximum inactivation rates ranged from −0.0296 ± 0.00845 (for *L. monocytogenes* grown at 37 °C on mixed salad) to −0.00772 ± 0.0106 (for *L. monocytogenes* grown at 21 °C on iceberg lettuce). In the case of *Salmonella*, the maximum inactivation rates ranged from −0.0328 ± 0.00718 (for cells grown at 4 °C on carrot) to −0.00796 ± 0.00791 (for cells grown at 37 °C on red cabbage). It should be noted that the calculated standard error rates are greater than or almost equal to the actual growth or inactivation rates. This could be attributed to the limited availability of experimental data during the short-term storage period. Further studies involving longer storage periods and more produce categories are warranted to better understand the effect of pre-growth temperatures on the survival kinetics of tested pathogens. 

## 4. Conclusions

This study investigated the effect of different pre-growth temperatures, 37, 21, and 4 °C on the subsequent growth, survival, and/or death kinetics of *L. monocytogenes* and *S. enterica* in fresh-cut produce during short-term simulated retail or consumer refrigerated storage conditions. Among the tested produce, mixed salad supported less the growth of *L. monocytogenes* but showed no effect on *S. enterica.* However, when pre-growth temperatures were changed to 21 and 4 °C, *L. monocytogenes* was found to be not affected (<1 log reduction), even in the mixed salad. Both *S. enterica* and *L. monocytogenes*, irrespective of pre-growth temperatures, survived well (<0.6 log reduction) in iceberg lettuce, red cabbage, and grated carrot during refrigerated storage. The findings of this study highlight the importance of considering the effect of appropriate pre-growth environmental conditions on the subsequent microbial survival kinetics in fresh-cut produce. This type of data would help to better estimate the risk in real-world scenarios and to develop robust interventions. Further studies should be focused on investigating the effect of other environmental stresses and the role of naturally present microbiota on the survival kinetics of foodborne pathogens across different fresh-cut produce commodities. 

## Figures and Tables

**Table 1:** Aerobic plate count (APC),and yeast and mold counts (log CFU/gm) in control samples during storage.

Type of Organism(s)	Time (h)	Iceberg Lettuce	Red Cabbage	Grated Carrot	Mixed Salad
APC	0	1.88 ± 1.26 ^a,A^	2.52 ± 0.56 ^a,A^	2.55 ± 0.25 ^a,A^	2.60 ± 0.50 ^a,A^
	12	2.25 ± 0.57 ^a,AB^	1.50 ± 0.91 ^a,B^	2.46 ± 0.29 ^a,AB^	2.72 ± 0.27 ^a,A^
	24	1.32 ± 0.83 ^a,A^	1.55 ± 1.00 ^a,A^	2.54 ± 0.32 ^a,A^	2.14 ± 0.64 ^a,A^
	48	2.02 ± 1.18 ^a,A^	1.65 ± 1.23 ^a,A^	2.47 ± 0.18 ^a,A^	2.24 ± 0.68 ^a,A^
	72	1.53 ± 1.01 ^a,A^	1.08 ± 0.86 ^a,A^	2.30 ± 0.47 ^a,A^	2.56 ± 0.40 ^a,A^
Yeast	0	4.09 ± 0.15 ^a,A^	3.99 ± 0.29 ^a,A^	4.02 ± 0.17 ^a,A^	4.08 ± 0.06 ^a,A^
	12	4.09 ± 0.09 ^a,A^	4.14 ± 0.07 ^a,A^	4.10 ± 0.05 ^a,A^	4.10 ± 0.03 ^a,A^
	24	4.15 ± 0.14 ^a,A^	4.15 ± 0.12 ^a,A^	4.16 ± 0.08 ^a,A^	4.12 ± 0.04 ^a,A^
	48	4.16 ± 0.05 ^a,A^	4.23 ± 0.05 ^a,A^	4.13 ± 0.12 ^a,A^	4.17 ± 0.16 ^a,A^
	72	4.06 ± 0.17 ^a,A^	4.02 ± 0.24 ^a,A^	4.08 ± 0.23 ^a,A^	4.14 ± 0.15 ^a,A^
Mold	0	0.66 ± 0.62 ^a,A^	0.96 ± 0.57 ^a,A^	0.94 ± 0.92 ^a,A^	0.90 ± 0.83 ^a,A^
	12	1.40 ± 0.55 ^a,A^	0.52 ± 0.74 ^a,A^	2.06 ± 0.44 ^ab,B^	1.52 ± 0.40 ^a,A^
	24	0.56 ± 0.76 ^a,A^	0.46 ± 0.64 ^a,A^	2.26 ± 0.23 ^ab,B^	1.46 ± 0.53 ^a,A^
	48	0.56 ± 0.76 ^a,A^	0.82 ± 0.75 ^a,A^	1.51 ± 1.38 ^ab,B^	1.49 ± 0.36 ^a,AB^
	72	0.40 ± 0.55 ^a,A^	0.72 ± 0.67 ^a,A^	2.07 ± 1.16 ^b,B^	1.94 ± 0.23 ^a,A^

The mean ± standard deviation is represented in the table (five replications). Lower-case letters within a row indicate a significant difference within a produce item during the storage period, and upper-case letters represent a significant difference among the different commodities at a single time point within a row.

**Table 2:** Parameter estimates of *L. monocytogenes* and *Salmonella enterica* survival kinetics in different produce using the Baranyi–Roberts model.

Type of Pathogen	Pre-Growth Temp ^a^ (°C)	Type of Produce	R^2^	SE of Fit ^b^	Initial Value (log CFU/g)	Maximum Rate ^c^ (1/h)
*L. monocytogenes*	37	Lettuce	0.817	0.0988	4.241 ± 0.0789	−0.00944 ± 0.0033
		Cabbage	0.967	0.0417	4.137 ± 0.0241	−0.018 ± 0.00275
		Carrot *	-	-	-	-
		Mixed salad	0.925	0.143	4.392 ± 0.13	−0.0296 ± 0.00845
	21	Lettuce	0.054	0.0998	4.174 ± 0.0982	−0.00772 ± 0.0106
		Cabbage	0.243	0.209	4.332 ± 0.209	−0.0213 ± 0.0243
		Carrot	0.657	0.119	4.0434 ± 0.0707	0.0122 ± 0.00734
		Mixed salad	0.952	0.0709	4.4 ± 0.0709	−0.052 ± 0.00956
	4	Lettuce	0.847	0.1	4.507 ± 0.1	−0.0291 ± 0.0119
		Cabbage	0.204	0.156	4.5 ± 0.156	−0.0184 ± 0.0191
		Carrot	0.653	0.18	4.0927 ± 0.165	−0.0166 ± 0.0108
		Mixed salad	0.847	0.1	4.507 ± 0.1	−0.0291 ± 0.0119
*S. enterica*	37	Lettuce	0.994	0.0215	6.0802 ± 0.0147	−0.0143 ± 0.00111
		Cabbage	0.194	0.199	5.372 ± 0.165	−0.00796 ± 0.00791
		Carrot	0.985	0.0575	6.0964 ± 0.0339	−0.03 ± 0.00349
		Mixed salad	0.92	0.106	6.136 ± 0.0983	−0.0296 ± 0.00702
	21	Lettuce	0.801	0.111	6.33 ± 0.0879	−0.00954 ± 0.00345
		Cabbage	0.919	0.101	5.843 ± 0.0933	−0.0279 ± 0.00667
		Carrot	0.95	0.106	6.318 ± 0.0723	−0.0245 ± 0.00549
		Mixed salad	0.957	0.0564	6.403 ± 0.0529	−0.0105 ± 0.00168
	4	Lettuce	0.876	0.102	6.269 ± 0.0794	−0.0101 ± 0.00301
		Cabbage	0.832	0.11	5.688 ± 0.0922	−0.0122 ± 0.00466
		Carrot	0.939	0.114	6.297 ± 0.0663	−0.0328 ± 0.00718
		Mixed salad	0.86	0.0993	6.225 ± 0.0909	−0.0163 ± 0.00599

^a^ Culture growth temperature prior to inoculation on different types of produce; ^b^ SE of fit, standard error of fit. ^c^ Maximum Rate: rate of inactivation (negative value) and growth (positive value). * Baranyi–Roberts model failed to fit the experimental data at these conditions.

## Data Availability

The data used to support the findings of this study can be made available by the corresponding author upon request.

## Supplementary Materials

The following supporting information can be downloaded at https://www.mdpi.com/article/10.3390/foods12234287/s1.
