# Supplementary material for: Effect of Different Pre-Growth Temperatures on the Survival Kinetics of Salmonella enterica and Listeria monocytogenes in Fresh-Cut Salad during Refrigerated Storage
Source: Foods. 2023 Nov 28;12(23):4287. doi: 10.3390/foods12234287 (PMC10706413; doi:10.3390/foods12234287)
Supplement: Supplementary file 1 [file foods-12-04287-s001.zip › foods-2649835-supplementary.pdf]

Table S1: Growth, survival, or death kinetics of *Listeria monocytogenes* (pre-cultured at 4 or 21 or 37ºC) on mixed salad, lettuce, red cabbage, and grated carrot during refrigerated storage (4 ºC) and 80 ±2 % RH for up to 72 h.

***4 ºC**		Log survival (CFU/cm^2) during refrigerated storage			
**Mixed**	**Time (h)**	**Rep-1**	**Rep-2**	**Rep-3**	**Mean**
	**0**	4.32	4.58	4.28	4.39
	**12**	4.2	4.28	3.88	4.12
	**24**	3.96	4.18	3.69	3.94
	**48**	3.54	4.03	3.45	3.67
	**72**	3.34	3.82	3.41	3.52
**Lettuce**	**Time (h)**				
	**0**	4.28	4.5	4.76	4.51
	**12**	3.93	4.2	4.32	4.15
	**24**	3.92	4.14	3.93	4.00
	**48**	3.75	3.97	4.32	4.01
	**72**	3.34	3.93	4.23	3.83
**Red cabbage**	**Time (h)**				
	**0**	4.28	4.89	4.34	4.50
	**12**	4.14	4.39	4.3	4.28
	**24**	4	4.69	3.93	4.21
	**48**	4.22	4.54	4.23	4.33
	**72**	3.98	3.95	4.12	4.02
**Carrot**	**Time (h)**				
	**0**	4.12	4.16	3.77	4.02
	**12**	4.03	4.11	3.99	4.04
	**24**	3.6	3.48	3.78	3.62
	**48**	3.37	3.38	3.52	3.42
	**72**	4.48	4.46	4.48	4.47
***21 ºC**					
**Mixed**	**Time (h)**	**Rep-1**	**Rep-2**	**Rep-3**	**Mean**
	**0**	4.27	4.55	4.38	4.40
	**12**	3.62	3.78	3.96	3.79
	**24**	3.46	4.12	3.64	3.74
	**48**	3.49	3.94	3.53	3.65
	**72**	3.06	4.18	3.55	3.60
**Lettuce**	**Time (h)**				
	**0**	3.4	4.32	4.72	4.15
	**12**	4.19	3.97	4.27	4.14
	**24**	3.53	4.14	4.06	3.91
	**48**	4.13	3.85	4.25	4.08
	**72**	3.88	3.89	4.2	3.99
**Red cabbage**	**Time (h)**				
	**0**	4.14	4.4	4.4	4.31
	**12**	4.3	4.25	3.88	4.14
	**24**	3.69	3.25	4.12	3.69
	**48**	3.67	4.37	4.24	4.09

	**72**	3.83	3.73	4.07	3.88
**Carrot**	**Time (h)**				
	**0**	3.82	4.24	3.86	3.97
	**12**	4.15	4.23	4.15	4.18
	**24**	4.34	3.82	3.78	3.98
	**48**	4.45	4.49	3.63	4.19
	**72**	4.48	4.44	4.5	4.47
***37 ºC**					
**Mixed**	**Time (h)**	Rep-1	Rep-2	Rep-3	Mean
	**0**	4.41	4.75	4.1	4.42
	**12**	4.11	4.19	3.69	4.00
	**24**	3.46	4.03	3.51	3.67
	**48**	3.37	3.39	3.39	3.38
	**72**	3.34	3.07	2.86	3.09
**Lettuce**	**Time (h)**				
	**0**	4.21	4.05	4.42	4.23
	**12**	4.18	4.15	4.31	4.21
	**24**	3.95	4.07	3.7	3.91
	**48**	3.81	3.96	3.75	3.84
	**72**	3.74	3.75	3.65	3.71
**Red cabbage**	**Time (h)**				
	**0**	4.05	4.02	4.43	4.17
	**12**	4.19	4.16	3.93	4.09
	**24**	4.11	4.28	4.05	4.15
	**48**	3.96	3.88	4.25	4.03
	**72**	3.56	3.72	3.56	3.61
**Carrot**	**Time (h)**				
	**0**	3.75	3.73	4.08	3.85
	**12**	4.13	4.15	4.28	4.19
	**24**	4.38	4.27	3.51	4.05
	**48**	4.55	4.6	3.56	4.24
	**72**	4.48	4.41	4.52	4.47

**L. monocytogenes* was pre-cultured at 4 or 21 or 37 ºC prior to inoculation on respective produce.

Table S2: Growth, survival, or death kinetics of *Salmonella enterica* (pre-cultured at 4 or 21 or 37ºC) on mixed salad, lettuce, red cabbage, and grated carrot during refrigerated storage (4 ºC) and 80 ±2 % RH for up to 72 h.

***4 ºC**		Log survival (CFU/cm2) during refrigerated storage			
**Mixed**	**Time (h)**	**Rep-1**	**Rep-2**	**Rep-3**	**Mean**
	**0**	6.84	5.28	6.39	6.17
	**12**	6.49	5.91	6.02	6.14
	**24**	5.29	5.9	6.15	5.78
	**48**	5.21	5.8	5.83	5.61
	**72**	5.9	5.38	5.71	5.66
**Lettuce**	**Time (h)**				
	**0**	6.86	5.46	6.38	6.23
	**12**	6.42	5.94	6.01	6.12
	**24**	6.4	5.93	6.12	6.15
	**48**	5.81	5.4	5.98	5.73
	**72**	5.48	5.41	5.85	5.58
**Red cabbage**	**Time (h)**				
	**0**	6.32	5.3	5.29	5.64
	**12**	6.08	5.8	5.09	5.66
	**24**	5.23	5.63	5.06	5.31
	**48**	5.18	5.38	4.91	5.16
	**72**	5.26	5.13	4.85	5.08
**Carrot**	**Time (h)**				
	**0**	6.6	5.67	6.24	6.17
	**12**	6.52	6.51	6.15	6.39
	**24**	6.49	6.33	6.16	6.33
	**48**	5.8	6.09	6.16	6.02
	**72**	4.53	5.26	5.95	5.25
***21 ºC**					
**Mixed**	**Time (h)**	**Rep-1**	**Rep-2**	**Rep-3**	**Mean**
	**0**	7.24	5.67	6.39	6.43
	**12**	6.55	6.18	6.15	6.29
	**24**	6.53	6.11	6.21	6.28
	**48**	6.06	5.99	6.08	6.04
	**72**	5.86	5.45	5.9	5.74
**Lettuce**	**Time (h)**				
	**0**	6.96	5.37	6.39	6.24
	**12**	6.57	6.13	6.15	6.28
	**24**	6.25	6.2	6.12	6.19
	**48**	5.88	5.43	6.12	5.81
	**72**	5.87	5.4	6.02	5.76

Red cabbage	Time (h)				
	0	6.2	5.6	5.58	5.79
	12	5.95	5.83	5.09	5.62
	24	5.14	5.23	5	5.12
	48	5.04	5.2	4.93	5.06
	72	5.13	5.18	4.77	5.03
Carrot	Time (h)				
	0	6.71	5.62	6.3	6.21
	12	6.56	6.67	6.01	6.41
	24	6.47	6.28	6.11	6.29
	48	5.27	6.16	5.98	5.80
	72	4.41	5.8	5.54	5.25
*37 ºC					
Mixed	Time (h)	Rep-1	Rep-2	Rep-3	Mean
	0	6.79	5.31	6.15	6.08
	12	6.15	5.71	5.85	5.90
	24	5.39	5.3	5.42	5.37
	48	5.23	5.21	5.41	5.28
	72	5.39	5.31	5.21	5.30
Lettuce	Time (h)				
	0	5.22	6.73	6.22	6.06
	12	5.91	6.41	5.98	6.10
	24	5.9	6.3	5.95	6.05
	48	5.7	5.78	5.88	5.79
	72	5.38	5.36	5.62	5.45
Red cabbage	Time (h)				
	0	5.31	5.16	5.25	5.24
	12	5.81	5.68	5.03	5.51
	24	5.12	5.19	4.99	5.10
	48	5.05	5	4.88	4.98
	72	5.16	4.98	4.81	4.98
Carrot	Time (h)				
	0	6.72	5.44	6.14	6.10
	12	6.55	5.97	5.94	6.15
	24	6.5	6.25	5.34	6.03
	48	5.85	6.05	5.34	5.75
	72	4.75	5.24	5.11	5.03

**S.enterica* was pre-cultured at 4 or 21 or 37 ºC prior to inoculation on respective produce.

Table S3: Growth, survival, or death kinetic parameter estimates of *L. monocytogens* pre-cultured at 37 or 21 or 4ºC and subsequently inoculated on different fresh produce during refrigerated storage.

[a]Temp (ºC)	[b]Empirical model	[c]Produce	[d]R^2	[e]SE of Fit	[f]Initial value (log CFU/g)	[g]Maximum Rate (1/h)
37	Baranyi and Roberts	Lettuce	0.817	0.0988	4.241 ± 0.0789	-0.00944 ± 0.0033
		Cabbage	0.967	0.0417	4.137 ± 0.0241	-0.018 ± 0.00275
		Carrot	-	-	-	-
		Mixed salad	0.925	0.143	4.392 ± 0.13	-0.0296 ± 0.00845
	Linear	Lettuce	0.846	0.0904	4.214 ± 0.0634	-0.00751 ± 0.00156
		Cabbage	0.708	0.124	4.23 ± 0.0872	-0.00704 ± 0.00215
		Carrot	0.742	0.117	3.937 ± 0.0819	0.00715 ± 0.00202
		Mixed salad	0.918	0.149	4.256 ± 0.104	-0.0174 ± 0.00258
21	Baranyi and Roberts	Lettuce	0.0539	0.0998	4.174 ± 0.0982	-0.00772 ± 0.0106
		Cabbage	0.243	0.209	4.332 ± 0.209	-0.0213 ± 0.0243
		Carrot	0.657	0.119	4.0434 ± 0.0707	0.0122 ± 0.00734
		Mixed salad	0.952	0.0709	4.4 ± 0.0709	-0.052 ± 0.00956
	Linear	Lettuce	-0.0163	0.103	4.108 ± 0.0725	-0.00173 ± 0.00179
		Cabbage	-0.0191	0.243	4.148 ± 0.17	-0.00404 ± 0.00421
		Carrot	0.628	0.124	3.971 ± 0.087	0.00598 ± 0.00215
		Mixed salad	0.454	0.239	4.105 ± 0.168	-0.00861 ± 0.00414
4	Baranyi and Roberts	Lettuce	0.847	0.1	4.507±0.1	-0.0291 ± 0.0119
		Cabbage	0.204	0.156	4.5± 0.156	-0.0184 ± 0.0191
		Carrot	0.653	0.18	4.0927 ± 0.165	-0.0166 ± 0.0108
		Mixed salad	0.847	0.1	4.507±0.1	-0.0291 ± 0.0119
	Linear	Lettuce	0.663	0.148	4.339±0.104	-0.00765 ± 0.00257
		Cabbage	0.513	0.122	4.419±0.0857	-0.00483 ± 0.00211
		Carrot	0.813	0.132	4.068 ± 0.102	-0.014 ± 0.00373
		Mixed salad	0.663	0.148	4.339±0.104	-0.00765 ± 0.00257

[a]Temperature (ºC): pre-growth temperatures of *L. monocytogens* prior to inoculation on fresh produce
[b]Empirical model: Different empirical models that fitted the experimental data using Combase DM Fit tool.
[c]Produce: Different types of fresh-cut produce tested.

[d]R^2: Standard error of coefficient. No value means the model failed to fit the data from that produce and storage condition.

[e]SE of fit, standard error of fit in Baranyi and Roberts or Linear analysis

[f]Initial value (δ): Starting concentration of bacteria on produce after the inoculation (Log CFU g^{-1})

[g] Maximum rate: Rate of reduction (negative value) or growth (positive value) in relation to the original inoculated population.

Table S4: Growth, survival, or death kinetic parameter estimates of *Salmonella enterica* pre-cultured at 37 or 21 or 4°C and subsequently inoculated on different fresh produce during refrigerated storage.

[a]**Temp (°C)**	[b]**Empirical model**	[c]**Produce**	[d]$\mathbf{R^2}$	[e]**SE of Fit**	[f]**Initial value (log CFU/g)**	[g]**Maximum Rate (1/h)**
37	Baranyi and Roberts	Lettuce	0.994	0.0215	6.0802 ± 0.0147	-0.0143 ± 0.00111
		Cabbage	0.194	0.199	5.372 ± 0.165	-0.00796 ± 0.00791
		Carrot	0.985	0.0575	6.0964 ± 0.0339	-0.03 ± 0.00349
		Mixed salad	0.92	0.106	6.136 ± 0.0983	-0.0296 ± 0.00702
	Linear	Lettuce	0.865	0.101	6.171 ± 0.0708	-0.00902 ± 0.00175
		Cabbage	0.409	0.171	5.341 ± 0.12	-0.00573 ± 0.00295
		Carrot	0.826	0.193	6.279 ± 0.135	-0.015 ± 0.00334
		Mixed salad	0.618	0.232	5.929 ± 0.163	-0.011 ± 0.00402
21	Baranyi and Roberts	Lettuce	0.801	0.111	6.33 ± 0.0879	-0.00954 ± 0.00345
		Cabbage	0.919	0.101	5.843 ± 0.0933	-0.0279 ± 0.00667
		Carrot	0.95	0.106	6.318 ± 0.0723	-0.0245 ± 0.00549
		Mixed salad	0.957	0.0564	6.403 ± 0.0529	-0.0105 ± 0.00168
	Linear	Lettuce	0.849	0.0973	6.31 ± 0.0682	-0.00815 ± 0.00168
		Cabbage	0.65	0.21	5.653 ± 0.147	-0.0105 ± 0.00363
		Carrot	0.798	0.213	6.463 ± 0.149	-0.0151 ± 0.00368
		Mixed salad	0.962	0.0531	6.445 ± 0.0372	-0.00926 ± 0.000918
4	Baranyi and Roberts	Lettuce	0.876	0.102	6.269 ± 0.0794	-0.0101 ± 0.00301
		Cabbage	0.832	0.11	5.688 ± 0.0922	-0.0122 ± 0.00466
		Carrot	0.939	0.114	6.297 ± 0.0663	-0.0328 ± 0.00718
		Mixed salad	0.86	0.0993	6.225 ± 0.0909	-0.0163 ± 0.00599
	Linear	Lettuce	0.916	0.0835	6.263 ± 0.0585	-0.00965 ± 0.00144

		Cabbage	0.822	0.113	5.64 ± 0.0794	-0.00866 ± 0.00196
		Carrot	0.638	0.277	6.456 ± 0.194	-0.0136 ± 0.00479
		Mixed salad	0.638	0.15	6.123 ± 0.105	-0.00803 ± 0.00259

[a]Temperature (ºC): pre-growth temperatures of *L. monocytogens* prior to inoculation on fresh produce
[b]Empirical model: Different empirical models that fitted the experimental data using Combase DM Fit tool.
[c]Produce: Different types of fresh-cut produce tested.
[d]R^2: Standard error of coefficient. No value means the model failed to fit the data from that produce and storage condition.
[e]SE of fit, standard error of fit in Baranyi and Roberts or Linear analysis
[f]Initial value (δ): Starting concentration of bacteria on produce after the inoculation (Log CFU g^{-1})
[g] Maximum rate: Rate of reduction (negative value) or growth (positive value) in relation to the original inoculated population.
